# A case report of two pelviscopic resections of fibrothecomas originating from the left ovary with recurrence after ten years

**DOI:** 10.1097/MD.0000000000034880

**Published:** 2023-08-18

**Authors:** Yun Sook Kim, H.J. Lee

**Affiliations:** a Department of Obstetrics and Gynecology, Cheonan-city, Chungnam, Korea; b Pathology, Soonchunhyang University College of Medicine, Soonchunhyang University Cheonan Hospital, Cheonan, Korea.

**Keywords:** fibrothecoma, pelviscopy, recurrence

## Abstract

**Rationale:**

Fibrothecomas are benign ovarians tumors. These are solid sex-cord-stromal tumors, accounting for 1% to 4.7% of all ovarian neoplasms. Their recurrence rate is known to be only 2% following ovarian sparing local mass excision. We report an uncommon case of 2 pelviscopic resections of fibrothecomas originating from the left ovary with recurrence after 10 years in a 34-year-old woman.

**Patient concerns:**

A 34-year-old married woman was diagnosed with 41 mm sized left ovarian recurrent fibrothecoma. We performed mass excision pelviscopically the first time 10 years ago. She gave birth to her second baby at 7 years after the first surgery. Ten years after the first surgery, fibrothecoma recurred on the same ovary with size larger than before.

**Diagnoses:**

At the time of its first occurrence 10 years ago, the ultrasound scan revealed a 34 × 23 mm-sized solid hypoechoic mass with well-demarcated margins and minimal Doppler flows. Ultrasound findings at the time of recurrence 10 years later showed the same findings, with its size increased to 41 × 40 mm. Final pathologic findings showed left ovarian fibrothecoma.

**Interventions:**

After her admission to the hospital, we performed pelviscopic removal of left ovarian fibroma. Microscopic examination revealed predominantly bland spindle cells with collagenous stroma, showing fascicular and storiform growth.

**Outcomes:**

Surgeries were successful. The patient had been followed-up regularly for 3 years after last surgery. She did not experience any complications. She remained disease-free.

**Lessons:**

Repetitive local mass excision appears to be an effective surgical option in women of reproductive age. Although there is a sufficient possibility of recurrence several years to decades after only mass excision, mass excision is more appropriate than total oophorectomy in women of childbearing age. Pelviscopic surgery is recommended.

## 1. Introduction

Fibrothecomas are solid benign tumors of ovary that account for 4% of all ovarian neoplasms.^[1]^ Ovarian fibrothecomas are composed of an admixture of fibrous and thecomatous elements. Histologically, these tumors are characterized by the presence of spindle, oval, or round cells forming various amount of collagen. They also contain a smaller proportion of theca cells.^[2]^ However, there is no universal agreement on which neoplasms should be classified as a fibrothecoma rather than either a fibroma or thecoma.^[3]^ Fibromas occur most frequently in women in their 50 seconds during perimenopause and postmenopause. They are hormonally inactive in most cases. Most of them are unilateral.^[4]^ Symptoms of fibrothecomas most commonly present due to mass effect causing compression on different organs. Torsion occurs in 8% of the patients.^[2]^ Given that the recurrence rate of ovarian fibroma/fibrothecoma following ovarian sparing local mass excision is 2%, local mass excision appears to be an effective surgical option for women of reproductive age.^[5]^ Here we report a very rare case of 2 pelviscopic resections of fibrothecoma originating from the left ovary with recurrence after 10 years with a literature review. This case report can be helpful for clinicians.

## 2. Case presentation

A 34-year-old Asian woman, gravida 2, para 2, presented with 41 mm sized left ovarian recurrent left ovarian fibrothecoma. She was 160 cm tall with a weight of 58 kg at the time of her visit. Her Papanicolaou smear done 3 months ago was normal.

**Figure 1.**
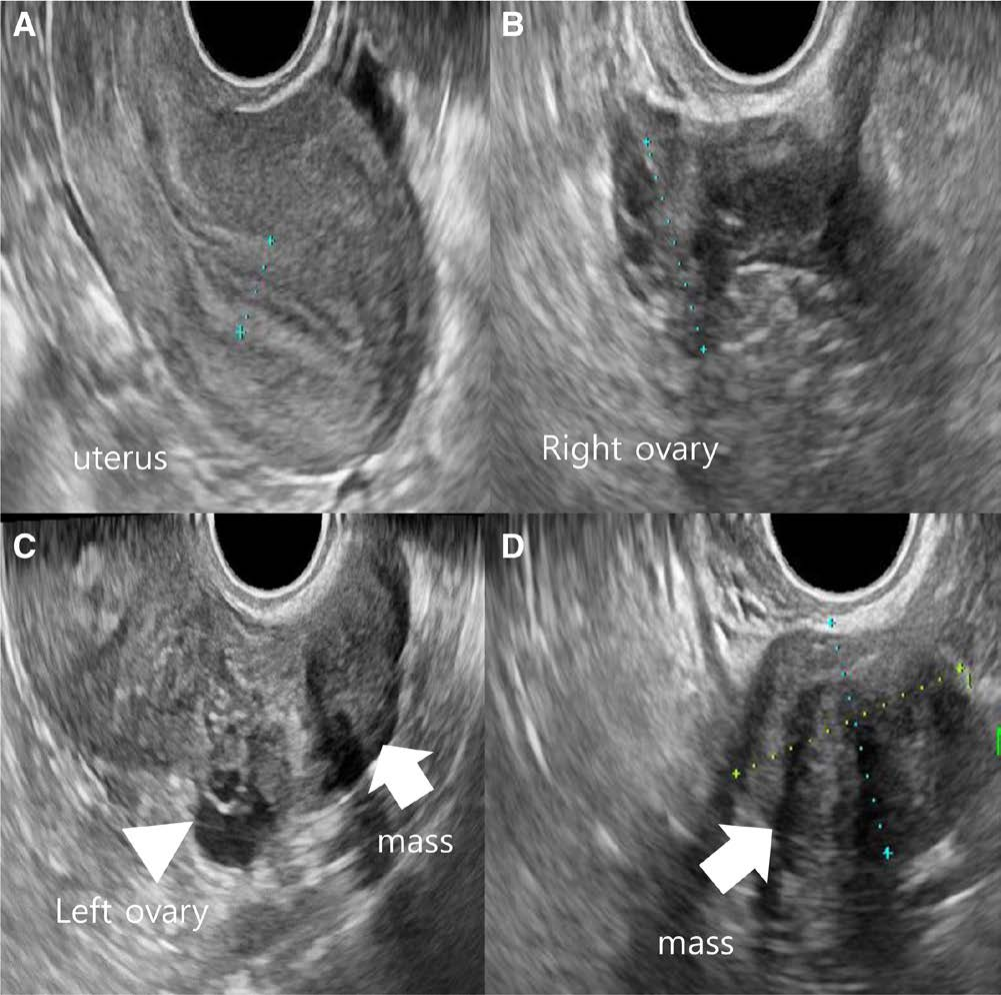
Transvaginal ultrasound findings. (A, B) The uterus and right ovary were normal on transvaginal ultrasound. (C) A well-defined, solid, oval, and hypoechogenic mass (arrow) with stripy shadows measuring 34 × 23 mm was seen next to the normal left ovary (arrow head). (D) There was no vascularity in the mass (arrow) on color Doppler.

**Figure 2.**
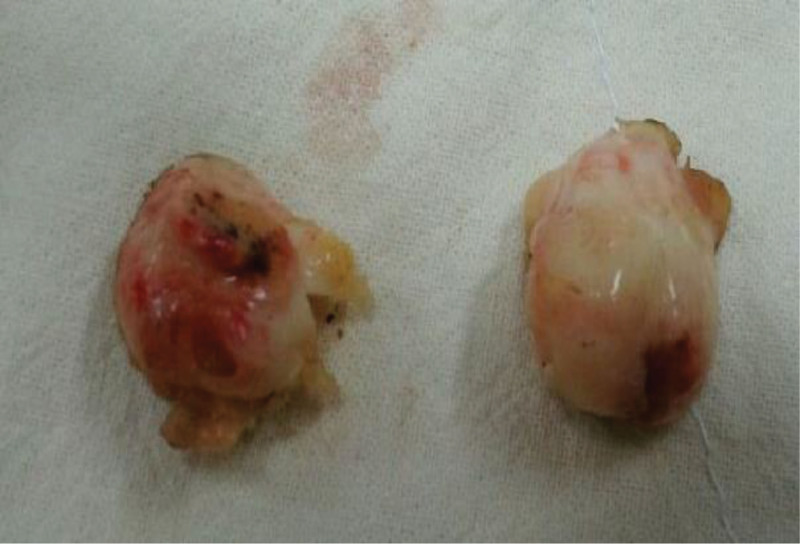
Gross examination of the specimen. The mass was measured to be 34 × 24 mm in size with two oval, solid, and grayish white fragments.

**Figure 3.**
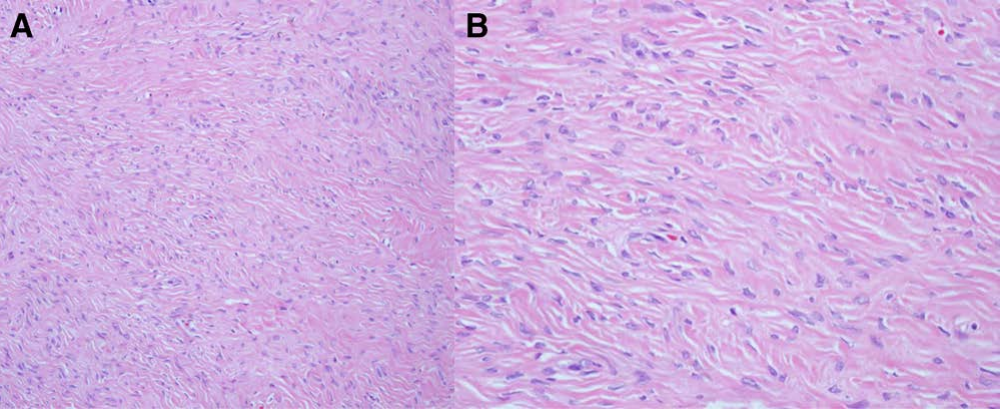
Microscopic findings. (A, B) Histological examination revealing predominantly bland spindle cells with collagenous stroma, showing fascicular and storiform growth (H & E, ×200, ×400). H&E = hematoxylin and eosin.

**Figure 4.**
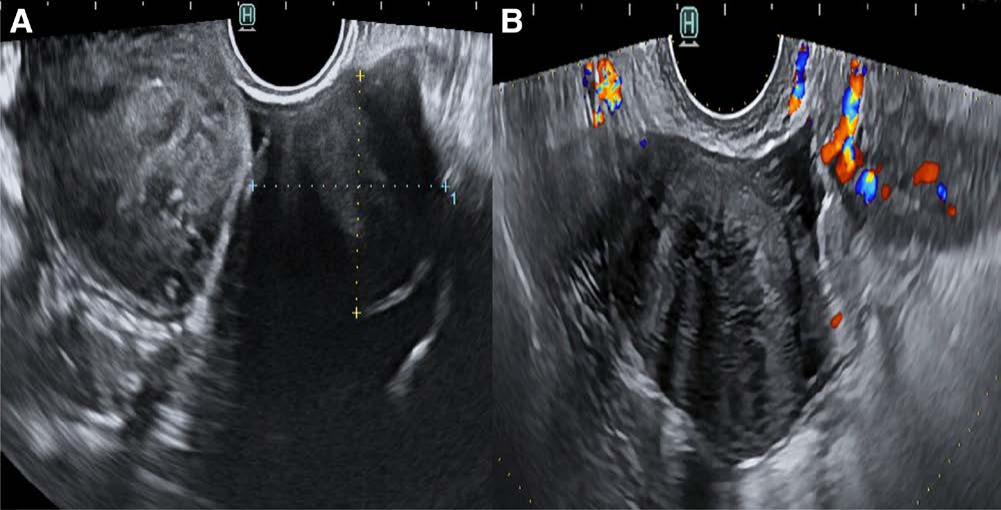
Transvaginal ultrasound findings. (A) A well-defined, solid, oval, and hypoechogenic mass with stripy shadows measuring 41 × 40 mm was seen in the left adnexa. (B) There was no vascularity in the mass on color Doppler.

**Figure 5.**
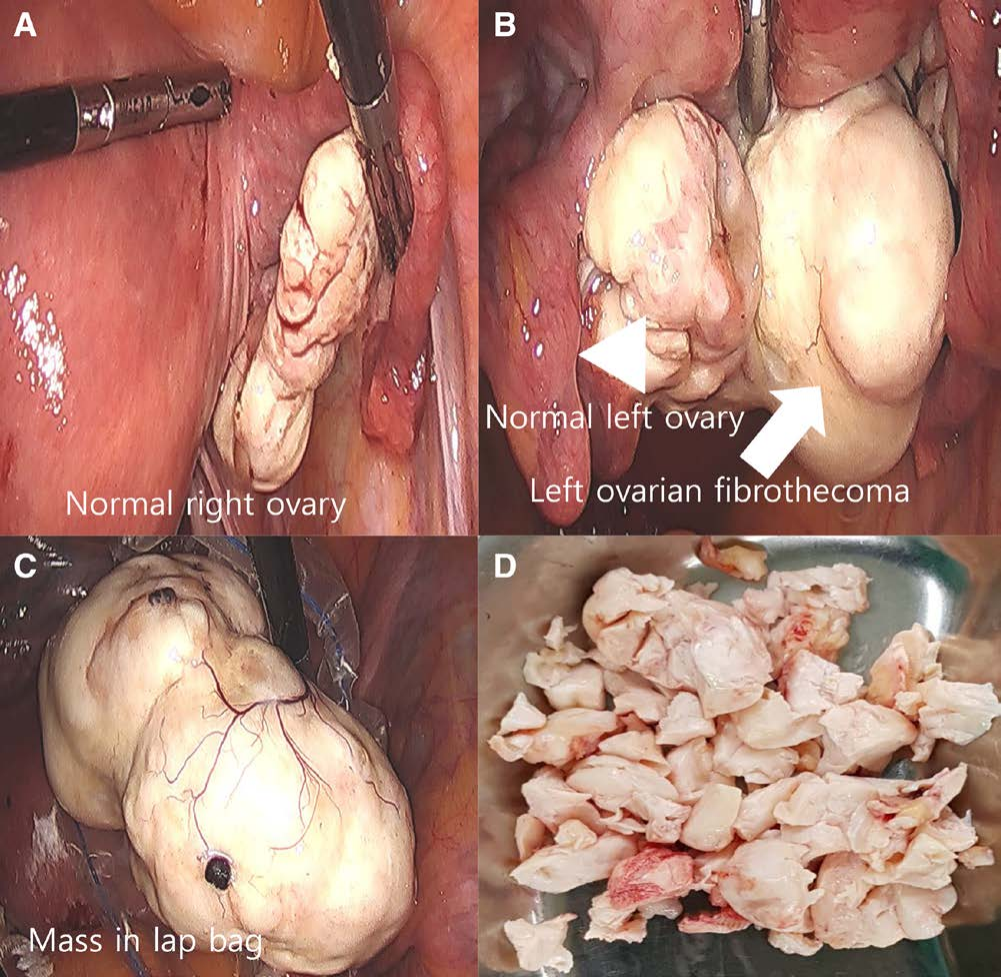
Pelviscopic findings and gross examination. (A) Pelviscopy showed normal right ovary. (B) The mass (arrow) was measured to be 42 × 40 × 36 mm in size. It was seen next to the normal left ovary (arrow head). (C) Putting the mass into the lap bag. (D) Several white pieces in cut surface.

She had ovarian surgery 10 years ago. At that time, her cancer antigen (CA) 125 (0–35 U/mL) level was 22.1 U/mL. All other laboratory values were within normal ranges. Gynecologic examination revealed no abnormalities of the vulva and cervix. Her uterus and right ovary were normal on transvaginal ultrasound. A well-defined, solid, oval, and hypoechogenic mass with stripy shadows measuring 34 × 23 mm was seen in the next to normal left ovary. There was no vascularity in the mass on color Doppler (Fig. 1A–D). We performed pelviscopic resection of the mass. On gross examination of the specimen, the mass was measured to be 34 × 24 mm in size with 2 oval and solid fragments (Fig. 2). Histological examination revealed predominantly bland spindle cells with collagenous stroma, showing fascicular and storiform growth in hematoxylin and eosin staining (Fig. 3A and B). Based on the above findings, the tumor was diagnosed as a fibrothecoma of left ovary. She gave birth to her second baby 7 years after the surgery. Ten years after the surgery, fibrothecoma recurred on the same ovary with size larger than before. This time her CA 125 level was 29 U/mL. Ultrasound findings at the time of recurrence 10 years later showed the same findings, with the size increased to 41 × 40 mm (Fig. 4A and B). We performed pelviscopic resection of the mass. On gross examination of the specimen, the mass was measured to be 42 × 40 × 36 mm in size (Fig. 5A–D). The patient has been followed-up regularly. She remains disease-free for 3 years after the surgery.

## 3. Discussions

Clinical-laboratory diagnosis of ovarian fibromas is not easy. Although ovarian fibromas are usually asymptomatic and diagnosed randomly, in about half cases (43.5%) the main symptom is abdominal pain, which is usually of low intensity.^[2]^ Acute abdominal pain that requires immediate treatment characterizes those cases in which the tumor has undergone torsion and necrosis. Elevated CA125 is rare. It is associated with Meigs’ syndrome. Solid nature of ovarian fibromas, their association with ascites and pleural effusion, and elevated serum CA125 levels require further diagnostic investigation to rule out ovarian malignancy.^[5]^ Transvaginal ultrasound and Doppler ultrasound imaging of the pelvis are important tools in preoperative diagnostic management of the disease. Typical sonographic features that support the diagnosis of ovarian fibromas include solid hypoechoic masses with well-demarcated margins and acoustic attenuation as well as minimal Doppler flow signals. A recent study has shown that the sensitivity and diagnostic accuracy of Doppler ultrasound for preoperative diagnosis of ovarian stromal tumors are higher than those of 2-dimensional ultrasound.^[6]^ Similarly, in our patient, the well-demarcated solid hypoechoic mass with minimal Doppler flow signals as imaged by Doppler ultrasonography strongly suspected the presence of ovarian fibroma/fibrothecoma. Computed tomography is difficult to distinguish ovarian fibroma from other ovarian masses. Magnetic resonance imaging is a second-line diagnostic method that has significantly contributed to improved preoperative diagnostic accuracy of ovarian fibromas.^[7]^ However, we believe that ultrasonographic diagnosis is almost accurate. Since the patient was a young woman without ascites, additional computed tomography or magnetic resonance imaging was not performed. Ovarian fibrothecomas can be misdiagnosed as uterine fibroids in about 40% of cases. Sometimes they are mistaken for malignant tumors of the ovary preoperatively.^[8]^ In the study of Cho et al^[1]^, the mean age of patients (n = 50) was 33.3 ± 6.9 years (range, 20–50 years), and the mean follow-up duration was 26.6 ± 19.2 months (range, 6–88 months). Fibroma was present in 40 patients, fibrothecoma in 7, and cellular fibroma in 3. Natural conception occurred in 11 of 12 patients who became pregnant during the follow-up period. On follow-up ultrasonography, 1 patient experienced recurrent disease at 50 months after the initial surgery, resulting in a crude overall recurrence rate of only 2%. Upon gross examination, ovarian sex-cord-stromal tumors are mostly solid masses with cut surface showing grayish white and yellowish areas.^[9]^ Histologically, fibrothecomas are composed of spindle-shaped fibroblastic cells that are collagen-producing and, hormonally inactive.^[10]^ Surgery is the treatment of choice. Based on the size, nature, location of the tumor, and patient’s age, it is possible to perform mass resection, especially in fertility-sparing treatment, a salpingo-oophorectomy or a radical hysterectomy in a case of tumor of multiple locations or uterine symptoms.^[11]^

## 4. Conclusion

Recurrence of ovarian fibrothecomas has rarely been reported. Management is guided by benign nature of the lesion. It consists of surgical removal of the mass. Preservation of the normal ovarian tissue is recommended, although there is risk of recurrence of the fibroma.

## Acknowledgements

The authors are grateful to Soonchunhyang University Cheonan Hospital for their assistance and encouragement. This study was supported by Soonchunhyang University Research Fund (20230009).

## Author contributions

**Conceptualization:** Yun Sook Kim.

**Data curation:** Yun Sook Kim.

**Investigation:** Yun Sook Kim.

**Writing – original draft:** Yun Sook Kim.

**Writing – review & editing:** Hyun Joo Lee.
